# Validation and Implementation of a Diagnostic Algorithm for DNA Detection of Bordetella pertussis, B. parapertussis, and B. holmesii in a Pediatric Referral Hospital in Barcelona, Spain

**DOI:** 10.1128/JCM.01231-18

**Published:** 2019-01-02

**Authors:** Ana Valero-Rello, Desiree Henares, Lesly Acosta, Mireia Jane, Iolanda Jordan, Pere Godoy, Carmen Muñoz-Almagro

**Affiliations:** a Institut de Recerca Sant Joan de Déu, Hospital Sant Joan de Déu, Barcelona, Spain; b Network of Epidemiology and Public Health, CIBERESP, Madrid, Spain; c Department of Statistics and Operations Research, Universitat Politecnica de Catalunya (BarcelonaTech), Barcelona, Spain; d Subdirecció General de Vigilància i Resposta a Alertas de Salut Pública (ASPCAT), Barcelona, Spain; e Pediatric Intensive Care Department, Hospital Sant Joan de Déu, Universitat de Barcelona, Barcelona, Spain; f Institut de Recerca Biomèdica de Lleida, Lleida, Spain; g School of Medicine, Universitat Internacional de Catalunya, Barcelona, Spain; University of Iowa College of Medicine

**Keywords:** *B. holmesii*, *B. parapertussis*, *Bordetella pertussis*, real-time PCR, whooping cough

## Abstract

This study aimed to validate a comprehensive diagnostic protocol based on real-time PCR for the rapid detection and identification of Bordetella pertussis, Bordetella parapertussis, and Bordetella holmesii, as well as its implementation in the diagnostic routine of a reference children’s hospital. The new algorithm included a triplex quantitative PCR (qPCR) targeting IS*481* gene (in B. pertussis, B. holmesii, and some Bordetella bronchiseptica strains), pIS*1001* (B. parapertussis-specific) and *rnase* P as the human internal control. Two confirmatory singleplex tests for B. pertussis (*ptxA*-Pr) and B. holmesii (hIS*1001*) were performed if IS*481* was positive. Analytical validation included determination of linear range, linearity, efficiency, precision, sensitivity, and a reference panel with clinical samples. Once validated, the new algorithm was prospectively implemented in children with clinical suspicion of whooping cough presenting to Hospital Sant Joan de Deu (Barcelona, Spain) over 12 months. Lower limits of detection obtained were 4.4, 13.9, and 27.3 genomic equivalents/ml of sample for IS*481* (on B. pertussis), pIS*1001* and hIS*1001*, and 777.9 for *ptxA*-Pr. qPCR efficiencies ranged from 86.0% to 96.9%. Intra- and interassay variabilities were <3% and <5%, respectively. Among 566 samples analyzed, B. pertussis, B. holmesii, and B. parapertussis were detected in 11.1%, 0.9% (only in females >4 years old), and 0.2% of samples, respectively. The new algorithm proved to be a useful microbiological diagnostic tool for whooping cough, demonstrating a low rate of other non-*pertussis* Bordetella species in our surveilled area.

## Abstract (precis)

This study aimed to validate a comprehensive diagnostic protocol based on real-time PCR for the rapid detection and identification of Bordetella pertussis, Bordetella parapertussis, and Bordetella holmesii, as well as its implementation in the diagnostic routine of a reference children’s hospital. The new algorithm included a triplex quantitative PCR (qPCR) targeting IS*481* gene (in B. pertussis, B. holmesii, and some Bordetella bronchiseptica strains), pIS*1001* (B. parapertussis-specific) and *rnase* P as the human internal control.

## INTRODUCTION

Pertussis is a vaccine-preventable acute respiratory disease primarily caused by Bordetella pertussis (1). Infants younger than 6 months are at higher risk to suffer from severe illness, hospitalization, and even fatal outcome (2, 3). Other, less prevalent Bordetella species, such as Bordetella parapertussis, Bordetella holmesii, and Bordetella bronchiseptica, can also produce pertussis-like illness.

In the last years and despite extensive vaccination programs, the resurgence of whooping cough has been documented worldwide. One possible explanation could be the replacement of B. pertussis by other Bordetella species (4, 5). This fact highlights the need of using precise diagnosis methods capable of identifying the etiological agent of the disease. An accurate identification at the species level is not only important from a clinical point of view to select the most appropriate antibiotic treatment, but also for health public purposes, since misdiagnosis of Bordetella species can lead to an incorrect assessment of pertussis vaccine effectiveness (6).

For this purpose, a number of microbiological techniques are readily available, such as culture, serology, and nucleic acid amplification tests (NAATs) (7). Although culture remains the gold standard, it has low sensitivity (8, 9). Serology is not an appropriate method to diagnose pertussis in pediatric populations, since it provides results that are difficult to interpret in immunized individuals and requires measuring antibody titers in the acute and convalescent phases of the disease, thus delaying time to result (10). Rapid, sensitive, and specific NAATs are being increasingly implemented to overcome the limitations of culture and serology (9, 11).

NAATs targeting IS*481*, at high copy number in the genome of B. pertussis, and IS*1001* for B. parapertussis are commonly used (12, 13). However, both targets are also present in B. holmesii and some B. bronchiseptica strains (14). Several algorithms combine nonspecific targets (IS*481*, IS*1001* or IS*1002*) and may include one or up to two specific targets for B. pertussis and B. holmesii (see Table S1). To our knowledge, only two published methods have reported the use of specific targets for the three most relevant Bordetella species (15, 16).

The present study aimed to adapt, optimize, and validate a diagnostic algorithm for the rapid detection and identification of B. pertussis, B. parapertussis, and B. holmesii. In addition, we sought to assess the disease burden caused by these species in our region by implementing the algorithm in pediatric patients suspected of pertussis.

(This work was presented in part at the 27th European Congress of Clinical Microbiology and Infectious Diseases, Vienna, Austria, 2017 [17].)

## MATERIALS AND METHODS

### Study design and setting.

Nasopharyngeal aspirates (NPAs) were prospectively collected from children and adolescents <18 years with clinical suspicion of whooping cough (according to CDC criteria), that were attended in Hospital Sant Joan de Deu (HSJD) between May 2016 and April 2017. This is a pediatric referral hospital that provides medical care services to more than 300,000 children in Catalonia (Spain). Information on age and sex variables of the patients was recorded for epidemiological purposes.

### Sample collection and DNA extraction.

Nasopharyngeal aspirates were processed according to the protocol established at the clinical laboratory of the study site (18). Specimens showing poor quality (*rnase* P > 35 cycle thresholds [*C_T_*]) or weak IS*481* positivity (40 > *C_T_* > 35) were subjected to additional DNA extraction using NucliSENS easyMag (bioMérieux, France), from an initial volume of 200 µl eluted into 25 µl.

### qPCR reference method.

The standardized laboratory method of Hospital Sant Joan de Déu (HSJD) consisted of a duplex quantitative PCR (qPCR) that included hydrolysis probes (Roche Diagnostics GmbH, Germany) targeting IS*481* and the human *rnase* P gene as a positive internal control for testing sample quality, as described in Brotons et al. (19) (Table 1). Delta Rn (dRn) *C_T_* values were manually set at 0.2 for both targets.

**TABLE 1:** List of oligonucleotides and probes used in the multiplex and confirmatory qPCRs

Target	Name^*d*^	Sequences^*c*^	Final concn (nM)	Reference or source
IS*481*	IS*481*-Ref Fwd	TCCGAACCGGATTTGAGAAAC	900	19
	IS*481*-Ref Rev	GTCGACGTAGGAAGGTCAATCG	900	
	IS*481*-Ref Probe	6-FAM-CGCCAACCCCCCAGTTCACTCA-TAMRA	300	
	IS*481* Fwd	CAAGGCCGAACGCTTCAT	770	15
	IS*481* Rev	GAGTTCTGGTAGGTGTGAGCGTAA	770	
	IS*481* Probe^*a*^	6-FAM-CAGTCGGCCTTGCGTGAGTGGG-BHQ1	260	
*rnase* P	*rnase* P Fwd	CCAAGTGTGAGGGCTGAAAAG	150	15
	*rnase* P Rev	TGTTGTGGCTGATGAACTATAAAAGG	150	
	*rnase* P Probe	YAK-CCCCAGTCTCTGTCAGCACTCCCTTC-BHQ1	250	
IS*1001*	pIS*1001* Fwd	TCGAACGCGTGGAATGG	600	15
	pIS*1001* Rev	GGCCGTTGGCTTCAAATAGA	600	
	pIS*1001* Probe^*b*^	Cy3-AGACCCAGGGCGCACGCTGTC-BHQ2	200	
	hIS*1001* Fwd	GGCGACAGCGAGACAGAATC	900	15
	hIS*1001* Rev	GCCGCCTTGGCTCACTT	900	
	hIS*1001* Probe^*b*^	Cy3-CGTGCAGATAGGCTTTTAGCTTGAGCGC-BHQ2	300	
*ptxA*-Pr	*ptxA*-Pr Fwd	CGCCAAGCTGAAGTAGCA	900	11
	*ptxA*-Pr Rev	AAGGAGCGTTCATGCCG	900	
	*ptxA*-Pr Probe	6-FAM-AGAATCGAGGGTTTTGTACGACGAATC-BBQ	300	This study

a Original fluorophore was modified for multiplexing.

b Original fluorophore and quencher were modified for multiplexing.

c Fluorophores and quenchers of hydrolysis probes are underlined. 6-FAM, 6-carboxyfluorescein; TAMRA, 6-carboxytetramethylrhodamine; BBQ, BlackBerry quencher; Cy3, cyanine 3; BHQ1, black hole quencher 1; BHQ2, black hole quencher 2; YAK, Yakima Yellow.

d Ref, reference; Fwd, forward; Rev, reverse.

Samples yielding an IS*481 C_T_* of <35 were considered probable B. pertussis isolates, inferred from the high copy numbers of such targets in this species (estimated in 50 to 200) (20). Samples with a *C_T_* value of 35 to 40 were reported as Bordetella spp., and as negative if *C_T_* was >40.

### New multiplex qPCR and diagnostic algorithm.

Previously published protocols for diagnosis of whooping cough were reviewed for designing the proposed algorithm (see Table S1). The method by Tatti et al. (15) was selected as the most complete and accurate basis for designing the new diagnostic algorithm, in relation to the number of species covered and the use of the human *rnase* P gene as the positive control (although not multiplexed). Its confirmatory target for B. pertussis, *ptxS1*, however, has been reported as cross-reactive with some strains of B. bronchiseptica (8, 21), so it was replaced by *ptxA*-Pr (11). The final design of the algorithm included three sequential qPCR assays for the specific identification of B. pertussis, B. parapertussis, and B. holmesii. The first triplex qPCR included the targets IS*481*, pIS*1001* (B. parapertussis-specific) (13, 15), and the human *rnase* P (Table 1). If IS*481* was positive, two confirmatory singleplex qPCRs were performed, *ptxA*-Pr for B. pertussis identification (21) and hIS*1001* for B. holmesii (Table 2) (22).

**TABLE 2:** Diagnostic algorithm used for the DNA detection and identification of *Bordetella* species

Species	qPCR result for			
	Multiplex^*a*^		Singleplex *ptxA-Pr*	Singleplex hIS*1001*
	IS*481*	pIS*1001*		
B. pertussis	+	−	+	−
B. parapertussis	−	+	−	−
B. holmesii	+	−	−	+
Bordetella spp.	+	−	−	−

a *rnase P* was used as the positive control. +, positive; −, negative.

Composition of the qPCR reactions only varied from the reference method in the concentrations and sequences of oligonucleotides, probes, and the reagents used for *rnase* P detection (Table 1). The *ptxA-Pr* probe was adapted by TIB-Molbiol (Berlin, Germany) to universal amplification conditions. dRn *C_T_* values were set at 0.2 for 6-carboxyfluorescein (6-FAM) or Yakima Yellow (YAK) probes and at 0.1 for cyanine 3 (Cy3).

### Analytical validation.

Bacterial strains used were B. pertussis CECT 7974, B. parapertussis ATCC 15311, and B. holmesii ATCC 51541. DNA standards were prepared from bacterial suspensions in phosphate-buffered saline solution (PBS). DNA was extracted using NucliSENS easyMag, and concentrations were quantified with the UV-visible (UV-Vis) spectrophotometer Q500 (Quawell, USA). Genome equivalents (GE) of standards were estimated assuming the molecular size of B. pertussis Tohama I (Genbank accession number BX470248; 4,086,190 bp), B. parapertussis Bpp5 (Genbank accession number HE965803; 4,800,120 bp), and B. holmesii ATCC 51541 (Genbank accession number CP007494; 3,699,670 bp).

DNA standards were freshly prepared, by 10-fold dilutions ranging from 10^6^ to 10° GE/ml of sample (10^4^ to 10^−2^ GE/reaction and 10^5^ to 10^−1^ fg DNA/reaction). Linear range and intra-assay variability were estimated by testing each dilution in triplicate on the same day, and consensus curves were used for calculating the efficiencies. Interassay variability was estimated with two additional replicates on successive days. Precision was acceptable if the mean coefficients of variation (CV) were ≤3% and ≤5% for intra-assay and interassay, respectively.

Analytical specificity was tested on a panel of 11 bacterial species, including Enterococcus faecalis, Escherichia coli, Haemophilus influenzae, Kingella kingae, Moraxella catarrhalis, Neisseria meningitidis, Pseudomonas aeruginosa, Staphylococcus aureus, Streptococcus agalactiae, Streptococcus mitis clinical isolates, and the Streptococcus pneumoniae R6.

In addition, a reference panel with clinical samples previously processed by the reference method was also tested by the new triplex qPCR. The purpose of this validation was to compare the performance of the two techniques targeting IS*481* (the gene shared by the two methods) in nasopharyngeal matrices. NPAs were collected between December 2015 and April 2016 from children <1 year old with suspected pertussis. All IS*481*-positive samples by the reference duplex qPCR (*n* = 22), in addition to 22 negative samples gathered during this period, were subsequently analyzed by the proposed algorithm. Samples were stored frozen at −80°C between both analyses.

### Statistical analysis.

Equations for the multiplex and singleplex qPCRs were calculated by plotting log10-transformed GE versus *C_T_* values. Lower limits of detection (LLOD) were estimated using probit regression analysis at 95% probability (see Table S2). Diagnostic sensitivity and specificity values were calculated as reported elsewhere (23).

Continuous variables were described as mean (standard deviation [SD]) or median values (interquartile range [IQR]). Parametric and nonparametric analyses were performed using a *t* test and the Mann-Whitney U test, respectively. Categorical data were analyzed by χ^2^ or Fisher’s exact test. Confidence intervals (CI) were set at 95% and significance at a two-sided *P* value of <0.05. All statistical analyses were performed with SPSS v.22 software (IBM Corp., USA), except for diagnostic sensitivity and specificity values, which were calculated with MedCalc Statistical Software v.17.6 (MedCalc Software Bvba, Belgium).

## RESULTS

### Analytical validation.

Linear ranges obtained spanned from 10^6^ to 10^1^ GE/ml of sample (*r*^2^ > 0.99). Efficiencies ranged from 86.0% to 96.9% (Table 3). LLOD for IS*481* were 4.4 and 60.3 GE/ml of sample for B. pertussis and *B. holmesii*, respectively. Target pIS*1001* showed a LLOD of 13.9 GE/ml of sample, and singleplex reactions hIS*1001* and *ptxA*-Pr showed LLOD of 27.3 and 777.9 GE/ml of sample, respectively (Table 3 and Table S2).

**TABLE 3:** New qPCR performances for each target and *Bordetella* species

qPCR type	Target	Bacteria	Target copy no.^*b*^	Curve^*a*^						Precision (CV %)	
				Linear range^*c*^	Slope^*d*^	Intercept	Efficiency (%)	*r^*2*^*	LLOD	Intra- assay	Interassay
Multiplex	IS*481*	B. pertussis	50 to 200	1.0 × 10^6^ to 1.0 × 10^1^	–3.58	40.55	90.3	0.999	4.4 × 10^0^	0.48	0.69
		B. holmesii	8 to 10	0.8 × 10^6^ to 0.8 × 10^2^	–3.71	44.36	86.0	0.999	6.0 × 10^1^	0.63	1.75
	pIS*1001*	B. parapertussis	∼20	2.2 × 10^6^ to 2.2 × 10^1^	–3.40	42.49	96.9	0.999	1.4 × 10^1^	0.32	0.58
Singleplex	*ptxA*-Pr	B. pertussis	1	1.0 × 10^6^ to 1.0 × 10^3^	–3.51	49.01	92.8	1.000	7.8 × 10^2^	0.94	1.35
Singleplex	hIS*1001*	B. holmesii	3 to 5	0.6 × 10^6^ to 0.6 × 10^2^	–3.50	43.72	92.9	0.998	2.7 × 10^1^	0.67	0.81

a Plotting *C_T_* versus log_10_ genome equivalents (GE) per ml of sample.

b Number of copies present in the genomes of the different *Bordetella* species (10).

c Genome equivalents of the specified bacteria per ml of sample. The DNA concentration range for each *Bordetella* species is as follows: *B. pertussis*, 0.9 × 10^5^ to 0.9 × 10^−1^ fg/reaction (2.0 × 10^4^ to 2.0 × 10^−2^ GE/reaction); *B. parapertussis*, 2.3 × 10^5^ to 2.3 × 10^−1^ fg/reaction (4.4 × 10^4^ to 4.4 × 10^−2^ GE/reaction); *B. holmesii*, 1.7 × 10^5^ to 1.7·10^−1^ fg/reaction (1.6 × 10^4^ to 1.6 × 10^−2^ GE/reaction) and 5.3 × 10^5^ to 5.3 × 10^−1^ fg/reaction (1.3 × 10^4^ to 1.3 × 10^−2^ GE/reaction) for multiplex and hIS*1001*, respectively.

d The symbol (−) indicates negative values. LLOD, lower limit of detection; CV, coefficient of variation.

Precision estimates (CV) were <3% for all reactions, with 0.32% to 0.94% and 0.58% to 1.75% values for intra-assay and interassay, respectively (Table 3). Results of the specificity panel showed the four Bordetella targets to be genus specific, and the confirmatory targets pIS*1001*, *ptxA*-Pr, and hIS*1001* were species specific.

The triplex qPCR correctly diagnosed all positive and negative samples of the reference panel of clinical samples. Sensitivity and specificity values for IS*481* were both 100% (95% CI, 84.6 to 100.0% and 83.2 to 100.0%, respectively). Mean *C_T_* for IS*481* in the reference and multiplex reactions were similar despite a freeze-thaw cycle between tests, with a mean difference between paired samples of 0.94 *C_T_* (95% CI, −0.40 to 2.78, *P* value = 0.159).

### Burden of disease caused by B. pertussis, B. parapertussis, and B. holmesii.

During the study period, 578 NPAs were collected, from which 9 were excluded, as they were either processed by a different technique (*n* = 2), invalid samples (*n* = 1), or did not meet age inclusion criteria (*n* = 6). In addition, 3 samples showed poor quality (*rnase* P > 35 *C_T_*) and were also disregarded. A total of 566 samples was finally included in the study. Of them, 484 (85.5%) were negative for the targets IS*481* or pIS*1001*. Eighty-two samples (14.5%) were Bordetella positive by either IS*481* (*n* = 81, 98.8%) or pIS*1001* (*n* = 1, 1.2%). The *ptxA-Pr* qPCR confirmed B. pertussis infection in 63 samples (76.8% of positives), and hIS*1001* identified B. holmesii in five samples (6.1% of positives), one of them also coinfected with B. pertussis. The remaining 13 positive samples (15.9%) could not be identified at the species level and were reported as Bordetella sp. infection (*C_T_* values = 30.65 to 38.17).

**FIG 1:**
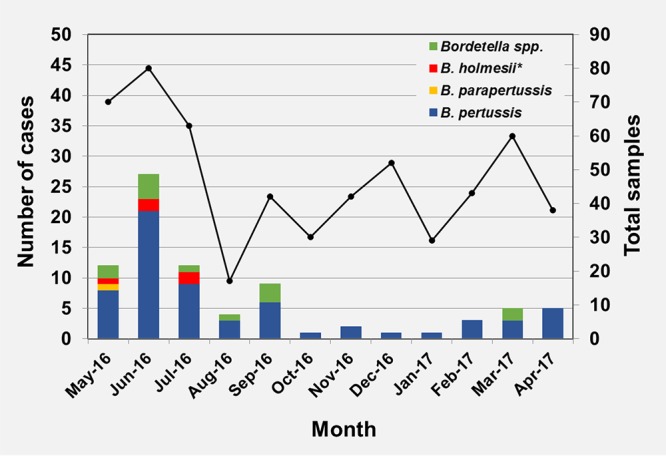
Seasonal distribution of microbiologically confirmed whooping cough cases by etiological agent (*n* = 566), includes one coinfection of B. holmesii and B. pertussis (*).

A seasonal distribution of positive samples was observed, showing a higher incidence during warmer months (*P* value* *<* *0.001) (Fig. 1), 62.2% of them identified within May to July (*n* = 51). B. pertussis was the most frequently detected species, while B. holmesii and B. parapertussis only circulated during the seasonal peak.

**FIG 2:**
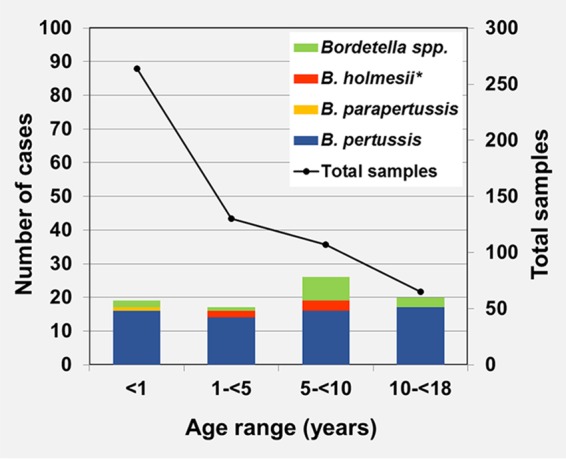
Age distribution of microbiologically confirmed cases and proportion of *Bordetella* species (*n* = 566). Includes one coinfection of B. holmesii and B. pertussis (*).

The median age of patients was 1.3 years (IQR, 0.24 to 5.85), and ages ranged from 7 days to 17.5 years. The distribution of B. pertussis was homogeneous across age groups, whereas B. holmesii was only detected in five children aged between 4.6 and 9.9 years, and B. parapertussis was only detected in an infant that was two months old (Fig. 2).

A remarkable difference in the positive rate was observed by age, with the infants younger than 1 year group showing the lowest positivity rate (7.2%). This rate increased with the age of patients (Fig. 2).

No differences in positivity rates were observed by gender, with proportions of 14.8% and 14.2% in males and females, respectively (*P* value = 0.857). In contrast, B. holmesii seemed to show a differential distribution, being only found in females (*n* = 5, *P* value = 0.06). B. parapertussis was only detected in a specimen taken from a male patient.

## DISCUSSION

The present study proposed a rapid and easy-to-use protocol based on three qPCR assays for specific DNA detection of B. pertussis, B. holmesii, and B. parapertussis. The multicopy targets IS*481*, pIS*1001*, and hIS*1001* were shown to be very sensitive, with LLOD values lower than 70 GE/ml of sample. The single-copy target *ptxA*-Pr had moderate but acceptable sensitivity, being able to detect <10^3^ GE/ml of sample. qPCR efficiencies were above 90.0% for all targets except for hIS*1001* (86%), all of them within the acceptability limits for qualitative methods (80.0% to 120.0%) (23). This simple and rapid multiplexed algorithm allowed the specific identification of the three Bordetella spp. in less than five hours.

The validated algorithm was implemented during 12 months at the study site. The most predominant species was B. pertussis, followed by B. holmesii and B. parapertussis. To date, there are few published European studies investigating the occurrence of other non-*pertussis* Bordetella infections. Retrospective studies performed in four European countries between 1992 and 2012 identified B. pertussis and B. parapertussis in 82.6 to 97.0% and 0.0 to 17.4% of the positive samples, respectively (5, 24–26). None of these investigations reported B. holmesii infections. However, two recent retrospective studies from 2013 to 2016 detected B. holmesii in 1.1 to 4.1% of positive samples and B. parapertussis in 0.3 to 8.2% of positive samples (27, 28). In addition, a study in France described a very high prevalence, up to 20%, of B. holmesii in adolescents and adults with pertussis-like symptoms (29). Our findings, in agreement with Mir-Cros et al. (28), confirm that B. holmesii is currently circulating in our region, and they could denote its increase as a causative agent of pertussis-like disease.

The incidence of pertussis in Spain has increased in all age groups despite the high levels of vaccination coverage (2). In our study, B. pertussis was shown to be evenly distributed among all ages, while B. holmesii was only detected in children >4 years old. This result is concordant with the higher prevalence of B. holmesii in symptomatic adolescents and adults that was previously reported in several studies (16, 29, 30).

A seasonal distribution of B. pertussis was observed, with higher occurrence in spring and summer, in line with the epidemiological trends of pertussis in Spain and Europe (31, 32). Although data of B. parapertussis and B. holmesii incidence rates presented in this study is limited, it appears to agree with literature suggesting cocirculation with B. pertussis, also supported by a remarkable number of B. pertussis-B. holmesii coinfections reported (16, 33, 34).

Bordetella infections were equally frequent in both sexes, as described by others (31). Interestingly, B. holmesii infection was only detected in females, although the low number of cases registered does not allow us to reach further conclusions on this potential association.

One study limitation was the lack of identification to species level in 15.9% of IS*481*-positive samples. Those Bordetella spp. likely corresponded to B. pertussis, in which *ptxA*-Pr qPCR results were negative due to low bacterial load or, less probably but possibly, to B. bronchiseptica strains for which no specific gene was investigated.

In conclusion, the new algorithm allowed improvement of accuracy of microbiological diagnosis of whooping cough at the study site by enhancing specificity while maintaining high sensitivity levels. In addition, we assessed the incidence of Bordetella spp. among the pediatric population of the geographical region of Catalonia during the algorithm implementation period. According to our data, the circulation of non-*pertussis* Bordetella species in this region seems to be minor and associated with seasonal increase of B. pertussis. Despite not representing a significant contribution to pertussis disease burden, it is essential to monitor the epidemiological patterns of these species to conduct an appropriate surveillance of the disease in our region. Nevertheless, our local data may not necessarily reflect the epidemiological status of Bordetella species in other areas, since differences in the species circulation can be highly influenced according to geographical and time variations (35). Therefore, local evaluations of diagnostic algorithms based on species-specific primers should carefully be undertaken before their implementation in any particular region.

### Ethical considerations.

This study was approved by the ethics committee of HSJD, in conformity with the Helsinki Declaration of 1975 (revised in 2000); the Spanish Organic Law 15/1999, on December 13th, on data protection; and law 14/2007, on July 3rd, on biomedical research. For the present study, no informed consent was requested, as this is a population-based study in which there were no activities that could compromise laboratory performance, and samples were duly anonymized.

## Supplementary Material

Supplemental file 1
