# Supplementary material for: Validation and Implementation of a Diagnostic Algorithm for DNA Detection of Bordetella pertussis, B. parapertussis, and B. holmesii in a Pediatric Referral Hospital in Barcelona, Spain
Source: J Clin Microbiol. 2019 Jan 2;57(1):e01231-18. doi: 10.1128/JCM.01231-18 (PMC6322476; doi:10.1128/JCM.01231-18)
Supplement: Supplemental file 1 [file 338605aaadc5ed658b224692c6521983_JCM.01231-18-s0001.pdf]

SUPPLEMENTAL MATERIAL

Table S1. Main features of molecular methods based on real-time PCR for detecting *Bordetella*.

Reference	Targets	IPC	Multiplex	Implemented
This study	**IS*481*** + **pIS*1001*** (Bpp) + *ptxA*-Pr (Bp) + hIS*1001* (Bh)	**Human**	Yes	Prospective
(1)	**IS*481*** + **IS*1001*** + **IS*1002*** + ***recA*** (Bh)	Not human	Yes (two)	No (retrospective)
(2)	**IS*481*** + **IS*1001*** + **hIS*1001*** (Bh) + *ptxA*[a] (Bp)	No	Yes	No (retrospective)
(3)	**IS*481*** + **IS*1001***	**Not human**	Yes	No
(4)	**IS*481*** + **BparaIS*1001*** (Bpp) + *ptxA*-Pr (Bp) + BholIS*1001* (Bh)	No	Yes	No (retrospective)
(5)	IS*481* + IS*1001* + PTpromoter (Bp)	Human	No	No (retrospective)
(6)	BP3385 + *ptxA*-Pr (Bp) + *recA* (Bh) + commercial (IS*481*+IS*1001*)	No	No	No (retrospective)
(7)	**IS*481*** + **IS*1001*** + **IS*1002***	**Not human**	Yes	No
(8)	IS*481* + **IS*1001***	**Not human**	Yes	No (retrospective)
(9)	**IS*481*** + **pIS*1001*** (Bpp) + **hIS*1001*** (Bh) + *ptxS1* (Bp[b])	Human	Yes	No (retrospective)
(10)	**IS*481*** + **IS*1001*** + ***ptxP*** (Bp)	No	Yes	No (retrospective)
(11)	IS*481* + *ptxA*-Pr (Bp)	Not human	No	Prospective
(12)	IS*481* + IS*1001*	Recommend	Yes	No
(13)	IS*481* + IS*1001*+ TPR (Bp)	No	No	No (retrospective)
(14)	**IS*481*** + **IS*1001***	No	Yes	No
(15)	**IS*481*** + IS*1001*	**Not human**	Yes	No (retrospective)
(16)	IS*481* + IS*1001* + *ptxA*-Pr (Bp)	Recommend	No	No

[a], only tested in some random samples; [b], *ptxS1* has been designed as specific target, however it has been reported cross-reactivity with some *B. bronchiseptica* isolates (16, 17). Specific targets are indicated by the *Bordetella* species abbreviation between parentheses. Targets included in multiplexed reactions are highlighted by bold letters. If two multiplex reactions, each combination appears underlined. Bp, *B. pertussis*; Bpp, *B. parapertussis*; Bh, *B. holmesii*; IPC, Internal positive control.

Table S2. Lower limit of detection and average Ct values of standard curves.

B. pertussis					*B. holmesii*						*B. parapertussis*		
**GE/mL[a]**	**Rep.**	**IS*481* (Ct)**	**Rep.**	***ptxA-Pr* (Ct)**	**GE/mL**	**Rep.**	**IS*481* (Ct)**	**GE/mL**	**Rep.**	**hIS*1001* (Ct)**	**GE/mL**	**Rep.**	**pIS*1001* (Ct)**
$1.0\cdot10^{6}$	5	19.12	5	28.19	$0.8\cdot10^{6}$	5	22.68	$0.6\cdot10^{6}$	5	23.39	$2.2\cdot10^{6}$	5	21.06
$1.0\cdot10^{5}$	5	22.71	5	31.77	$0.8\cdot10^{5}$	5	26.39	$0.6\cdot10^{5}$	5	26.74	$2.2\cdot10^{5}$	5	24.37
$1.0\cdot10^{4}$	5	26.20	5	35.17	$0.8\cdot10^{4}$	5	30.10	$0.6\cdot10^{4}$	5	30.13	$2.2\cdot10^{4}$	5	27.76
$1.0\cdot10^{3}$	5	29.87	10	38.40	$0.8\cdot10^{3}$	5	33.84	$0.6\cdot10^{3}$	5	33.46	$2.2\cdot10^{3}$	5	31.05
$1.0\cdot10^{2}$	5	33.39	10	nd	$0.8\cdot10^{2}$	10	38.07	$0.6\cdot10^{2}$	10	37.12	$2.2\cdot10^{2}$	5	34.54
$1.0\cdot10^{1}$	10	37.73	5	nd	$0.8\cdot10^{1}$	10	nd	$0.6\cdot10^{1}$	10	nd	$2.2\cdot10^{1}$	10	38.24
$1.0\cdot10^{0}$	10	nd	5	nd	$0.8\cdot10^{0}$	5	nd	$0.6\cdot10^{0}$	5	nd	$2.2\cdot10^{0}$	10	nd
**LLOD (GE/ml)**		$4.4\cdot10^{0}$		$7.8\cdot10^{2}$	**LLOD (GE/ml)**		$6.0\cdot10^{1}$			$2.7\cdot10^{1}$	**LLOD (GE/ml)**		$1.4\cdot10^{1}$

[a] Genome equivalents (GE) per mL of sample. Rep., number of replicates. Ct, cycle threshold. nd, not detected. Average Ct values were calculated from the replicates at each concentration.

References:

1. Martini H, Detemmerman L, Soetens O, Yusuf E, Piérard D. 2017. Improving specificity of Bordetella pertussis detection using a four target real-time PCR. PLoS One 12:e0175587.
2. Pittet LF, Emonet S, François P, Bonetti E-J, Schrenzel J, Hug M, Altwegg M, Siegrist C-A, Posfay-Barbe KM. 2014. Diagnosis of whooping cough in Switzerland: differentiating Bordetella pertussis from Bordetella holmesii by polymerase chain reaction. PLoS One 9:e88936.
3. Arbefeville S, Levi MH, Ferrieri P. 2014. Development of a multiplex real-time PCR assay for the detection of Bordetella pertussis and Bordetella parapertussis in a single tube reaction. J Microbiol Methods 97:15–19.
4. Rodgers L, Martin SW, Cohn A, Budd J, Marcon M, Terranella A, Mandal S, Salamon D, Leber A, Tondella ML, Tatti K, Spicer K, Emanuel A, Koch E, McGlone L, Pawloski L, Lemaile-Williams M, Tucker N, Iyer R, Clark TA, Diorio M. 2013. Epidemiologic and laboratory features of a large outbreak of pertussis-like illnesses associated with cocirculating Bordetella holmesii and Bordetella pertussis - Ohio, 2010-2011. Clin Infect Dis. Oxford University Press.
5. Grogan JA, Logan C, O'Leary J, Rush R, O'Sullivan N. 2011. Real-time PCR-based detection of Bordetella pertussis and Bordetella parapertussis in an Irish paediatric population. J Med Microbiol 60:722–729.
6. Njamkepo E, Bonacorsi S, Debruyne M, Gibaud SA, Guillot S, Guiso N. 2011. Significant finding of Bordetella holmesii DNA in nasopharyngeal samples from French patients with suspected pertussis. J Clin Microbiol 49:4347–4348.
7. Roorda L, Buitenwerf J, Ossewaarde JM, Van Der Zee A. 2011. A real-time PCR assay with improved specificity for detection and discrimination of all clinically relevant Bordetella species by the presence and distribution of three insertion sequence elements. BMC Res Notes 4:11.
8. Slinger R, Moldovan I, Hyde L, Chan F. 2011. Rapid detection of Bordetella pertussis and Bordetella parapertussis in clinical and molecular proficiency panel specimens with a novel intercalating dye-based PCR assay. Scand J Infect Dis 43:968–971.
9. Tatti KM, Sparks KN, Boney KO, Tondella ML. 2011. Novel multitarget real-time PCR assay for rapid detection of Bordetella species in clinical specimens. J Clin Microbiol 49:4059–4066.
10. Xu Y, Xu Y, Hou Q, Yang R, Zhang S. 2010. Triplex real-time PCR assay for detection and differentiation of Bordetella pertussis and Bordetella parapertussis. APMIS 118:685–691.
11. Fry NK, Duncan J, Wagner K, Tzivra O, Doshi N, Litt DJ, Crowcroft N, Miller E, George RC, Harrison TG. 2009. Role of PCR in the diagnosis of pertussis infection in infants: 5 Years' experience of provision of a same-day real-time PCR service in England and Wales from 2002 to 2007. J Med Microbiol 58:1023–1029.
12. Guillot S, Guiso N, Riffelmann M, Wirsing Von König CH. 2014. Laboratory Manual for the Diagnosis of Whooping cough caused by Bordetella pertussis/ Bordetella parapertussis. World Heal Organ 1–41.
13. Knorr L, Fox JD, Tilley P a G, Ahmed-Bentley J. 2006. Evaluation of real-time PCR for diagnosis of Bordetella pertussis infection. BMC Infect Dis 6:1–12.
14. Kösters K, Reischl U, Schmetz J, Riffelmann M, Wirsing von König CH. 2002. Real-time LightCycler PCR for detection and discrimination of Bordetella pertussis and Bordetella parapertussis. J Clin Microbiol 40:1719–1722.
15. Templeton KE, Scheltinga SA, Van der Zee A, Diederen BMW, Kruijssen AM, Goossens H, Kuijper E, Claas ECJ. 2003. Evaluation of real-time PCR for detection of and discrimination between Bordetella pertussis, Bordetella parapertussis, and Bordetella holmesii for clinical diagnosis. J Clin Microbiol 41:4121–4126.

16. Dalby T. 2012. Guidance and protocol for the use of real- time PCR in laboratory diagnosis of human Guidance and protocol for the use of RT-PCR for diagnosis of Bordetella infections. ECDC.

17. Kolodkina V, Martinov V, Babenko A. 2014. Multiplex real-time PCR assay for detection and differentiation of Bordetella pertussis and Bordetella parapertussis. Iran J Microbiol 6:140–8.
